# Ohio State Dental Society

**Published:** 1881-01

**Authors:** 


### OHIO STATE DENTAL SOCIETY.

The thirteenth annual meeting of the Ohio State Dental Society was held in Columbus on the 7th, 8th and 9th of December, and was one of the best meetings of that body ever held.

There were not many papers read, and but three of the eight subjects on the programme discussed, but they were well and thoroughly discussed. A far larger number of the members than ever before taking part in the discussion.

The papers presented were good. A large amount of important miscellaneous business was transacted, thus showing the main scope of the aim and operations of the Society.

The Society is free from debt and has quite a sum in its treasury.

Dr. Geo. Watt was present, and added much to the interest of the occasion. By the special request of the Society he gave a lecture, occupying one hour and ten minutes in its delivery, upon the decay of the teeth; especially describing the agents of decay and their mode of action.

The improvement in Dr. Watt's health was a very agreeable surprise to all his friends, who had not seen him for some time, and the great hope and desire of all is that he may be fully restored.

There is now promise of greater usefulness and efficiency of this society than for a number of years past.

